# Pathogenic alpha-synuclein aggregates preferentially bind to mitochondria and affect cellular respiration

**DOI:** 10.1186/s40478-019-0696-4

**Published:** 2019-03-14

**Authors:** Xinhe Wang, Katelyn Becker, Nathan Levine, Michelle Zhang, Andrew P. Lieberman, Darren J. Moore, Jiyan Ma

**Affiliations:** 1 Center for Neurodegenerative Science, Van Andel Research Institute, 333 Bostwick Avenue N.E, Grand Rapids, MI 49503 USA; 2 Department of Pathology, University of Michigan Medical School, Ann Arbor, MI48109 USA

**Keywords:** α-Synuclein, Aggregation, PFF, Mitochondria, Lewy body, Parkinson’s disease, α-Synucleinopathies

## Abstract

Misfolded alpha-synuclein (αSyn) is a major constituent of Lewy bodies and Lewy neurites, which are pathological hallmarks of Parkinson’s disease (PD). The contribution of αSyn to PD is well established, but the detailed mechanism remains obscure. Using a model in which αSyn aggregation in primary neurons was seeded by exogenously added, preformed αSyn amyloid fibrils (PFF), we found that a majority of pathogenic αSyn (indicated by serine 129 phosphorylated αSyn, ps-αSyn) was membrane-bound and associated with mitochondria. In contrast, only a minuscule amount of physiological αSyn was mitochondrial bound. In vitro, αSyn PFF displayed a stronger binding to purified mitochondria than did αSyn monomer, revealing a preferential mitochondria binding by aggregated αSyn. This selective mitochondrial ps-αSyn accumulation was confirmed in other neuronal and animal αSyn aggregation models that do not require exogenously added PFF and, more importantly, in postmortem brain tissues of patients suffering from PD and other neurodegenerative diseases with αSyn aggregation (α-synucleinopathies). We also showed that the mitochondrial ps-αSyn accumulation was accompanied by defects in cellular respiration in primary neurons, suggesting a link to mitochondrial dysfunction. Together, our results show that, contrary to physiological αSyn, pathogenic αSyn aggregates preferentially bind to mitochondria, indicating mitochondrial dysfunction as the common downstream mechanism for α-synucleinopathies. Our findings suggest a plausible model explaining the formation and the peculiar morphology of Lewy body and reveal that disrupting the interaction between ps-αSyn and the mitochondria is a therapeutic target for α-synucleinopathies.

**Electronic supplementary material:**

The online version of this article (10.1186/s40478-019-0696-4) contains supplementary material, which is available to authorized users.

## Introduction

Parkinson’s disease (PD) is a common neurodegenerative disorder in the elderly and its characteristic motor symptoms result from the loss of dopaminergic neurons in the substantia nigra [11, 37]. Mitochondrial dysfunction is sufficient to cause nigral dopaminergic neuron loss [11], which is consistent with the idea that these neurons are selectively vulnerable to various toxic insults [49]. Besides mitochondria dysfunction, genetic studies have established a causative role of dominant mutations or multiplication of *SNCA* (αSyn gene) in familial PD [8, 23, 38, 45] and the association of *SNCA* polymorphism with a higher risk of idiopathic PD [4], demonstrating a key contribution of αSyn to PD pathogenesis. αSyn is an intrinsically disordered protein and is abundantly expressed in neurons [18, 23]. During the disease, αSyn is subjected to a variety of post-translational modifications [2]. Among them, the phosphorylation at serine 129 is most relevant to the pathogenic process and is commonly used to indicate the pathogenic αSyn species [1, 14].

In addition to PD, αSyn aggregation is also a common pathological hallmark for a group of neurodegenerative diseases known as α-synucleinopathies, which include PD, dementia with Lewy bodies (DLB), and multiple system atrophy (MSA) [15, 17]. A large body of evidence supports that the aggregation process of αSyn, including both oligomerization and amyloid fibril growth, is closely related to the pathogenesis of α-synucleinopathies [23]. Notably, both increased αSyn expression by *SNCA* multiplication [8, 45] and the presence of disease-associate αSyn mutations can increase the aggregation propensity of αSyn [10]. Reducing αSyn clearance is also able to increase the amount of αSyn and thereby enhances αSyn aggregation and neurotoxicity [33]. Moreover, inoculating preformed αSyn amyloid fibrils (PFF) into wild-type mice induces endogenous αSyn aggregation and subsequent nigral dopaminergic neuron degeneration [29], demonstrating that αSyn aggregation is sufficient to cause neurodegeneration. Despite these advances, very little is known about the detailed mechanism of how αSyn aggregation causes neurotoxicity and contributes to the pathogenic process.

It has been reported that αSyn may play a role in the physiology and/or pathology of mitochondrial function [50]. Even though αSyn does not have a mitochondrial targeting sequence, several groups reported its localization in mitochondria or mitochondria-associated membranes (MAMs) [9, 12, 16, 25–27, 44, 47], and showed that αSyn affects a variety of mitochondrial functions, from Ca^2+^ signaling, to complex I activity, to mitochondrial morphology and dynamics [50]. However, given the well-established presynaptic localization of αSyn and its role in synaptic vesicle release [5–7, 18, 19, 21], it remains unclear how much physiological αSyn is mitochondria-associated and to what extent it affects mitochondrial function. Moreover, whether the mitochondrial αSyn localization contributes to the disease process and how it is related to the other major pathogenic event, αSyn aggregation, are completely unknown.

Some recent studies started to explore the potential relationship between pathogenic αSyn species and mitochondria. Using a proximity ligation assay (PLA), Di Maio et al. showed that αSyn oligomer and S129E phosphomimic mutant each bind TOM20 on the mitochondrial outer membrane and impair mitochondrial protein import [13]. Using exogenously added αSyn oligomer, Ludtman et al. showed mitochondrial localization of αSyn oligomer by PLA and the impairment of mitochondrial function [28]. They also showed that increased endogenous αSyn aggregates in neurons derived from an *SNCA* triplication patient were also in the vicinity of mitochondrial ATP synthase, indicating a mitochondrial localization [28]. Using human pluripotent stem cells expressing mutant *SNCA*, Ryan et al. reported fragmented mitochondria and the clustering of αSyn aggregates in the mitochondria of *SNCA* mutant neurons, but not in the isogenic control neurons [42]. The methodologies used in above studies, including transient transfection, exogenously added αSyn oligomers, *SNCA* mutant cells, and heavy dependence on imaging-based analyses, make it difficult to conclude how much pathogenic αSyn is associated with mitochondria; whether mitochondrial association is a main pathogenic pathway for αSyn aggregates; whether the mitochondrial ps-αSyn accumulation occurs in neurons that express wild-type αSyn at endogenous level, and more importantly, whether such accumulation occurs in α-synucleinopathy patients.

To understand the cellular mechanism of αSyn aggregation and the resulting neurotoxicity, we adopted a highly reproducible primary neuron model developed by Volpicelli-Daley et al., in which the neuronal accumulation of ps-αSyn was induced by exogenously added PFF [52]. We took an unbiased approach to study the subcellular localization of ps-αSyn and found that the majority of ps-αSyn was associated with mitochondria. This finding was verified in other neuronal and mouse models and was also confirmed with postmortem brain tissues from α-synucleinopathy patients. Consistent with these findings, our in vitro results showed a preferential binding of mitochondria by aggregated αSyn. Moreover, we have also showed that the mitochondrial accumulation of ps-αSyn is associated with mitochondrial respiration defects, suggesting mitochondrial dysfunction as a downstream consequence of aggregated αSyn.

## Materials and methods

### Purification of recombinant αSyn and preparation of αSyn PFF

Mouse αSyn was purified as previously described [3, 51]. Purified αSyn was dialyzed against PBS buffer (VWR#97062–732), aliquoted, and stored at − 80 °C. When needed, aliquots of αSyn were thawed, concentrated to 350 μM, and shaken at 37 °C for 7 d at 1000 rpm to prepare mature mouse αSyn preformed fibrils (PFF) [51]. PFF was aliquoted and stored at − 80 °C. All PFF preparations were verified by the thioflavin T fluorescence assay and by imaging using a Tecnai G2 Spirit TWIN transmission electron microscope. The purification of human αSyn and the preparation of hPFF were performed with the same procedures described above.

### Primary neuronal culture

P1 mouse or rat cortical neurons were isolated and cultured essentially according to a previously published protocol [51]. At 7 d in vitro (DIV), neurons were treated with or without 140 nM αSyn monomer or PFF. Treated neurons were cultured as usual and collected after 8–9 d or after a specified time. PFF was sonicated before each usage with a water-bath cup-horn sonicator (Misonix XL2020) at 50% power for 5 min. For proteasomal inhibition, primary neurons were treated with DMSO or 100 nM epoxomicin for 8 h, and after incubation, cell lysates or homogenates were prepared.

### Immunofluorescence staining and proximity ligation assay (PLA)

**Fig. 1:**
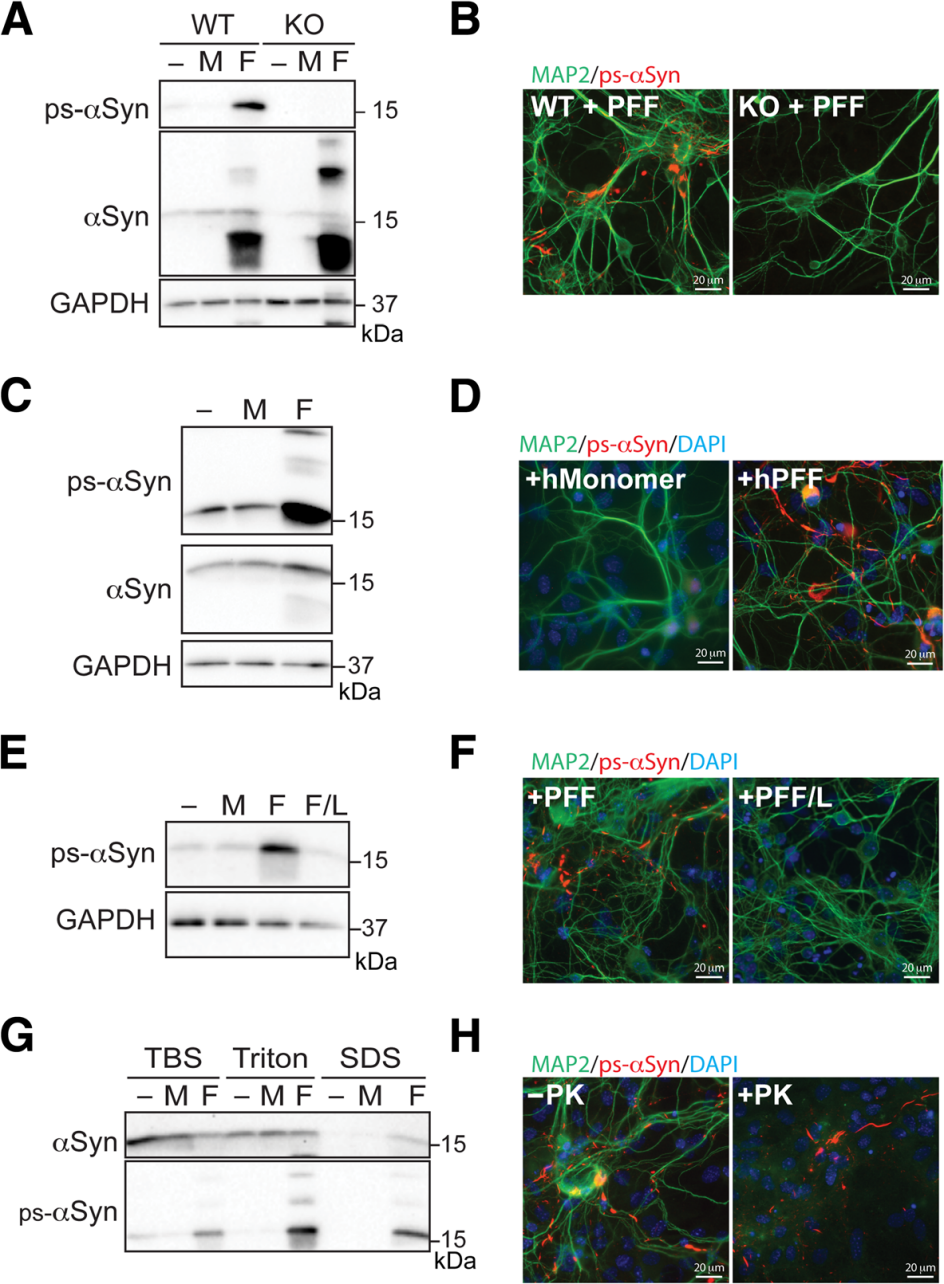
PFF treatment induces ps-αSyn in primary neurons. **a, b** Primary cortical neurons derived from wild-type (WT) and αSyn knockout (KO) mice were untreated (−) or treated with αSyn monomer (M) or PFF (F) as indicated. **c, d** Primary cortical neurons derived from OVX mice were untreated (−) or treated with human αSyn monomer (M) or PFF (F). **e, f** Primary neurons derived from wild-type mice were untreated (−) or treated with αSyn monomer (M), αSyn PFF (F), or lysozyme PFF (F/L). ps-αSyn (in **a**, **c**, and **e**) and total αSyn (in **a** and **c**) were detected by immunoblot analysis and GAPDH was used as a loading control. In **b**, **d**, and **f**, ps-αSyn was visualized by immunofluorescence staining (red), and MAP2 stain (green) was used as a neuronal marker. **g** Primary neurons, untreated (−) or treated with αSyn monomer (M) or PFF (F), were sequentially extracted with TBS, 1% Triton/TBS, and 2% SDS/TBS. The presence of ps-αSyn and total αSyn was detected by immunoblot analysis. **h** PFF-treated neurons were incubated with or without a 10-min 10 μg/mL PK digestion at 37 °C prior to immunofluorescence staining to detect ps-αSyn (red) and MAP2 (green)

Neurons at 16 DIV were fixed with 4% formaldehyde in culture medium at 37 °C for 15 min and then permeabilized with 0.2% Triton-100 for 10 min at room temperature. PLA staining was performed with anti-TOM20 (Abcam, 1:100) and anti-αSyn phospho (Ser129) (Abcam, 1:5000) using Duolink In Situ Detection Reagents (Sigma# DUO92008), following the protocol provided by the manufacturer. For immunofluorescence staining, anti-MAP2 (Sigma, 1:250), anti-αSyn phospho (Ser129)(Abcam, 1:1000), and anti-TOM20 (Abcam 1:100) were used as primary antibodies. Alexa 488-conjugated goat anti-mouse IgG and Alexa 594-conjugated goat anti-rabbit IgG (ThermoFisher) were used as secondary antibodies. Images were visualized with an Olympus IX83 microscope and a Nikon A1plus-RSi scanning confocal microscope. The Pearson’s correlation coefficient was calculated with Nikon Elements Analysis AR5.11.00 (Nikon). For the proteinase K digestion experiment in Fig. 1h, fixed neurons were incubated with 10 μg/mL proteinase K at 37 °C for 10 min before the immunofluorescence staining.

### Sequential extraction

At DIV 16, neurons were homogenized into TBS buffer (50 mM Tris, 150 mM NaCl, pH 7.5), sonicated at 50% power for 5 min (Misonix XL2020), and centrifuged at 100,000×*g* for 30 min at 4 °C. The pellets were resuspended in 1% Triton-100 in TBS, sonicated, and centrifuged as described above. The supernatant was collected as the Triton-soluble fraction. The pellet was resuspended in 2% SDS in TBS.

### Differential centrifugation to separate mitochondrial and cytosolic/microsomal fractions

Primary cortical neurons were washed once with ice-cold neuronal membrane buffer (NMB, 250 mM sucrose, 20 mM HEPES, 10 mM KCl, 1.5 mM MgCl_2_, 1 mM EGTA, 1 mM EDTA, and 1 mM DTT, pH 7.5) and then were homogenized in NMB plus proteinase and phosphatase inhibitors (NMB+). Dissected spinal cords of transgenic mice, or brain tissue from patients were directly homogenized in NMB+. The homogenates were centrifuged at 600 × *g* (for cells) or 700×*g* (for tissue) at 4 °C for 5 min to remove nuclei and unbroken cells. The supernatants were used as the postnuclear supernatant (PNS). To separate mitochondrial and cytosolic/microsomal fractions, the PNS was centrifuged at 10,000×*g* for 5 min at 4 °C. The supernatants were collected as cytosolic/microsomal fractions, and the pellets (the mitochondrial fraction) were resuspended in NMB+.

### Density gradient

For the iodixanol gradient analysis, either PNS or isolated mitochondria were mixed with iodixanol stock solution to reach the final concentration of 36%. The mixture (0.8 mL) was loaded to the bottom of the ultracentrifuge tube. On top of it, 0.8 mL of 31% and 0.8 mL of 10% iodixanol were sequentially laid. After centrifugation at 52,000×*g* for 4 h at 4 °C (ThermoFisher rotor S55-S), 0.2 mL fractions were collected from top to bottom. Discontinuous sucrose gradient analysis was performed as previously described [53].

### Western blots

Samples were separated on 16% SDS polyacrylamide gel or pre-made 4–20% gradient SDS polyacrylamide gel (ThermoFisher) and transferred to PVDF membranes (GE Healthcare). The membranes were fixed with 4% paraformaldehyde and 0.1% glutaraldehyde for 30 min at room temperature and probed with various antibodies. The primary antibodies were anti-αSyn phospho (Ser129) (Abcam 51,253, 1:2500), anti-αSyn (BD Biosciences clone 42, 1:2500), anti-ATPIF1 (ThermoFisher, 1:1500), anti-ATP5A (Abcam, 1:1000), anti-GAPDH (Stressgen, 1:1000), anti-Calnexin (Sressgen, 1:1000), and anti-Syntaxin 6 (Sigma, 1:1000). The HRP-conjugated goat anti-mouse or anti-rabbit IgG antibodies (Bio-Rad) were used as the secondary antibodies. Signals were developed by ECL 2 substrate (Pierce) and scanned with ChemiDoc (Bio-Rad). The signal intensity was measured with the FIJI software.

### Mitochondrial stress test

Primary cortical neurons were seeded in Agilent Seahorse 96-well plate at 1.0 × 10^4^ per well. At DIV 7, neurons were treated with either αSyn monomer or PFF. At DIV 14, the oxygen consumption rate was measured with Seahorse XF^e^96 (Agilent) after sequentially adding 1 μM oligomycin, 1.25 μM FCCP (carbonyl cyanide-p-trifluoromethoxyphenylhydrazone)**,** and 0.5 μM rotenone/antimycin A (XF Cell Mito Stress Test Kit from Agilent).

### Animals

C57BL/6J mice were from Van Andel Institute internal colony. CD (Sprague Dawley) IGS rats were obtained from Charles River. Transgenic mice expressing human A53T αSyn from the mouse prion protein promoter (line G2–3, kindly provided by Dr. Michael Lee, University of Minnesota) were described previously [24]. The bilateral intramuscular PFF injection was performed according to a previously published protocol [43] with slight modification. Briefly, the PFF used for the intramuscular injection was prepared by shaking 350 μM αSyn at 1000 rpm for 2 d at 37 °C. PFF was aliquoted and stored at − 80 °C. Before each injection, PFF was diluted to 0.1 μg/μL and sonicated with the water-bath cup-horn sonicator (Misonix XL2020) at 25% power for 2 h, and then 10 μL of PFF (0.1 μg/μl) or 10 μL PBS was injected into biceps femoris on each hindlimb.

### Statistical analysis

Statistical analyses were performed with GraphPad Prism software version 6.05.

## Results

### PFF-induced ps-αSyn in primary neurons

We first characterized the PFF-induced synucleinopathy model in primary neurons. Our results showed that adding PFF (Additional file 1: Figure S1) was sufficient to induce the phosphorylation of endogenous αSyn and no ps-αSyn was detected in PFF-treated primary neurons derived from αSyn knockout mice (Fig. 1a). Immunofluorescence staining verified that ps-αSyn was accumulated in the neurons labeled with the neuronal marker MAP2 (Fig. 1b). Using an antibody detecting total αSyn, we found that the majority of αSyn in PFF-treated wild-type or αSyn knockout neurons was cleaved (Fig. 1a). Because the only αSyn in the knockout neurons was from PFF, this result suggested that the majority of the exogenously added PFF was truncated in neurons. The induction of ps-αSyn was specific to the addition of αSyn PFF; adding the same amount of αSyn monomer did not produce ps-αSyn. Moreover, the exogenously added monomer was barely detectable by immunoblot analysis after 9 days (Fig. 1a, monomer in αSyn knockout neurons), suggesting a more rapid clearance than that of exogenously added PFF. The same result was obtained with rat primary neurons (Additional file 1: Figure S2).

To determine whether the findings can be reproduced with human αSyn, we cultured neurons derived from the BAC transgenic mice (OVX mice) that express human wild-type αSyn at twice the endogenous level of mouse αSyn and on a mouse αSyn knockout background [20]. These neurons were either untreated or treated with human αSyn monomer or PFF prepared with human αSyn (hPFF). Immunoblot analysis (Fig. 1c) revealed that ps-αSyn was induced in neurons treated with hPFF, but not in neurons untreated or treated with human αSyn monomer. Immunofluorescence staining confirmed that ps-αSyn was in neurons (Fig. 1d).

To ensure that the observed effects were not because of toxic effects of the amyloid fibrils, we compared αSyn PFF with fibrils of another amyloidogenic protein, lysozyme (Fig. 1e and f). The ps-αSyn was accumulated only in αSyn PFF-treated neurons, not in those treated with lysozyme fibrils, suggesting the ps-αSyn was likely resulted from seeding by αSyn PFF.

We also determined the aggregation status of ps-αSyn by sequential detergent extraction. ps-αSyn was enriched in Triton- and SDS-soluble fractions, whereas total αSyn in untreated cells appeared mainly in the TBS-soluble fraction (Fig. 1g). Immunofluorescence staining revealed that PFF-induced ps-αSyn resisted proteinase K digestion (Fig. 1h). These results led us to conclude that the majority of PFF-induced ps-αSyn in primary neurons was aggregated.

### Preferential binding of PFF-induced ps-αSyn to mitochondria

**Fig. 2:**
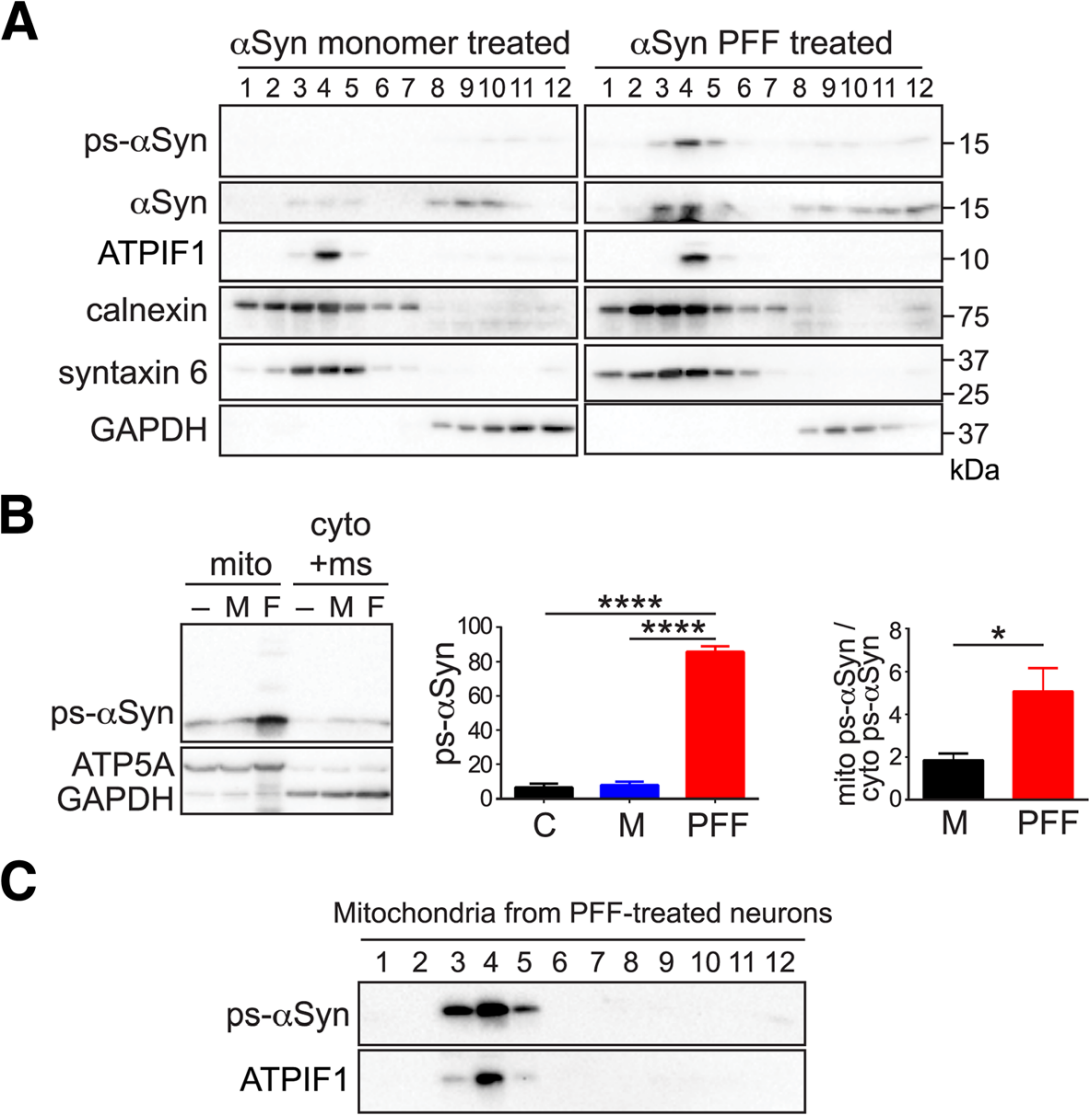
Accumulation of PFF-induced ps-αSyn in mitochondria. **a** PNS prepared from αSyn monomer-treated or PFF-treated primary neurons were subjected to iodixanol gradient separation. The presence of each protein was detected by immunoblot analyses. **b** PNS from untreated (−) or αSyn monomer (M)- or PFF (F)-treated rat primary neurons were subjected to differential centrifugation to separate the mitochondrial and cytosolic/microsomal fractions, which were verified by immunoblot analyses with antibodies against mitochondrial ATP synthase (ATP5A) and cytosolic GAPDH, respectively. The ps-αSyn in these fractions was detected by immunoblot analysis. The bar graph in the middle shows the average ± standard error of mitochondrial ps-αSyn from five independent experiments. “C” represents control. Statistical significance was determined by one-way ANOVA followed by Dunnett’s multiple comparisons test (F = 322.8; *n* = 5; *p* < 0.0001). The bar graph on the right shows the ratio of ps-αSyn in the mitochondria over ps-αSyn in the cytosolic/microsomal fraction, which was the average ± standard error of three independent experiments. Statistical significance was determined by paired *t*-test (*n* = 3; *p* = 0.048). * represents *p* < 0.05. **c** The mitochondria isolated from PFF-treated neurons were subjected to iodixanol gradient separation, and the presence of ps-αSyn and the mitochondrial marker ATPIF1 was detected by immunoblot analyses

To determine the subcellular localization of ps-αSyn, postnuclear supernatant (PNS) was prepared from neurons treated with αSyn monomer or PFF and subjected to iodixanol density-gradient separation (Fig. 2a). The majority of total αSyn in monomer-treated neurons remained at the bottom, co-migrating with the cytosolic marker GAPDH (glyceraldehyde 3-phosphate dehydrogenase). The ps-αSyn in PFF-treated neurons, however, migrated to the upper, membrane-bound fractions. Among membrane markers, ps-αSyn appeared to be concentrated in the peak fraction of mitochondrial marker ATPIF1 (Fig. 2a), which is similar to the finding using a discontinuous sucrose density gradient (Additional file 1: Figure S3) [53]. These results led us to hypothesize that the PFF-induced ps-αSyn may be mitochondria-bound.

We tested this possibility by separating the cell homogenates into mitochondrial and cytosolic/microsomal fractions by differential centrifugation. We found that majority of ps-αSyn was indeed in the mitochondrial fraction (Fig. 2b and Additional file 1: Figure S4). To rule out the possibility that the appearance of ps-αSyn in mitochondria fraction was simply due to its aggregation status, we subjected isolated mitochondria to a density gradient that was optimized for mitochondria purification. The ps-αSyn co-migrated with mitochondrial marker ATPIF1 to the top of the gradient (Fig. 2c), confirming its association with the membranous mitochondria.

**Fig. 3:**
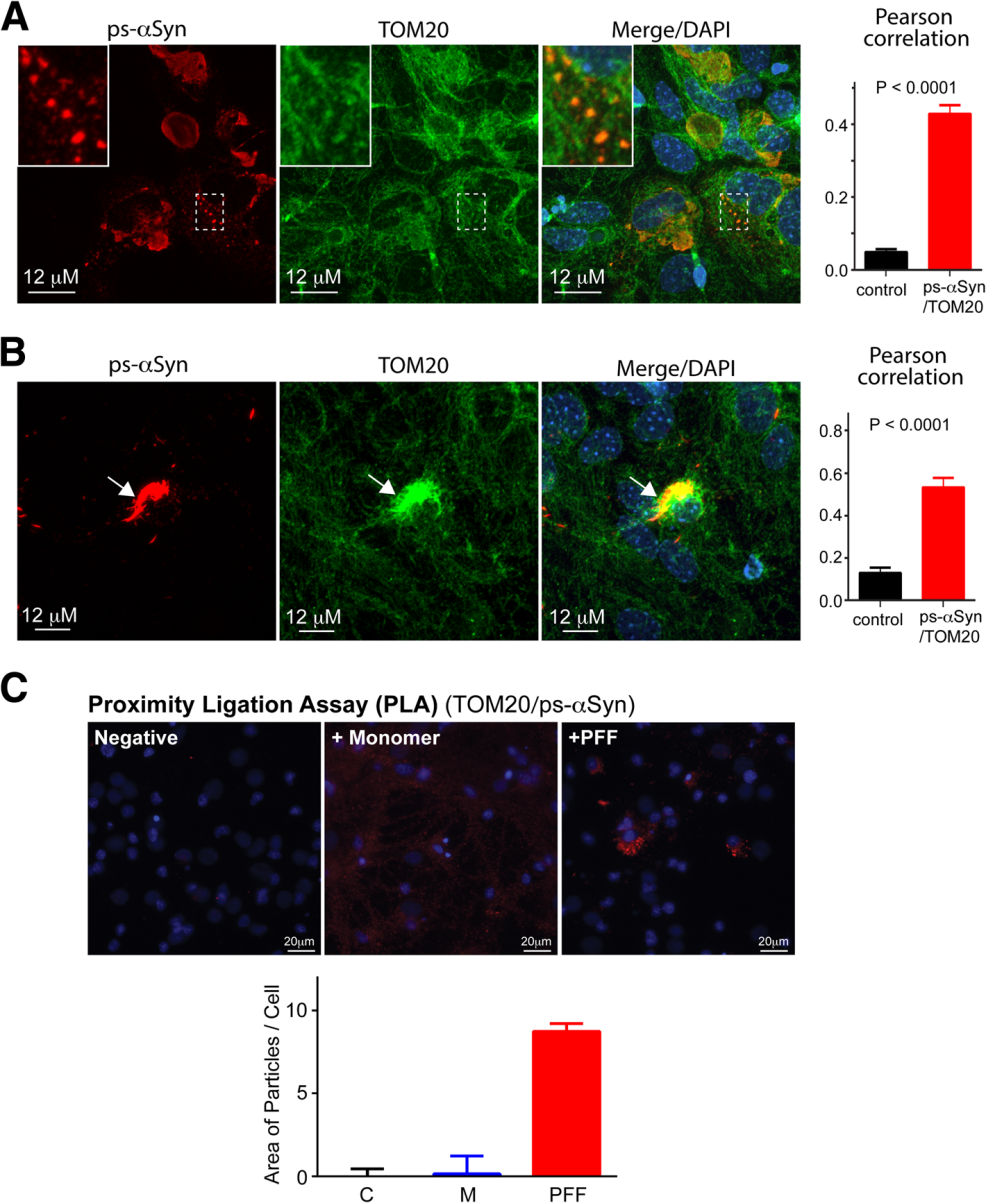
Co-localization of PFF-induced ps-αSyn with mitochondria marker TOM20. **a** Primary cortical neurons were prepared from OVX mice, cultured for 7 days, then treated with hPFF (PFF prepared with human αSyn), cultured for another 9 d, and subjected to immunofluorescence staining with antibodies against ps-αSyn and TOM20. Inserts are enlarged images of the areas indicated by the dashed boxes. **b** Mouse primary neurons were treated with PFF and subjected to immunofluorescence staining with antibodies against ps-αSyn and TOM20. Arrows point to the area with strong ps-αSyn accumulation, but weak TOM20 staining. The Pearson correlation coefficient in **a** and **b** was determined from 12 individual cells. Statistic difference was determined by an unpaired t-test (*n* = 12; p < 0.0001). **c** A proximity ligation assay (PLA) with antibodies against ps-αSyn and TOM20 was performed in primary neurons treated with αSyn monomer or PFF. Representative images include left, negative control (omitting anti-TOM20 antibody); center, αSyn monomer-treated neurons; right, PFF-treated neurons. The bar graph shows average ± standard error of three independent experiments with six areas quantified per experiment. Statistical analysis was performed with one-way ANOVA followed by Dunnett’s multiple comparisons test (F = 66.40; *n* = 6; p < 0.0001)

Immunofluorescence staining was used to determine the subcellular localization of PFF-induced ps-αSyn. Compared with ps-αSyn in PFF-treated mouse primary neurons (Fig. 3b), the human ps-αSyn in hPFF-treated OVX neurons appeared to be less clumpy or less tightly packed (Fig. 3a), which presumably reflects the high propensity of mouse αSyn to aggregate [41]. Nevertheless, co-localization of ps-αSyn with the mitochondrial outer membrane protein TOM20 was detected in both hPFF-treated OVX neurons (Fig. 3a) and in PFF-treated mouse neurons (Fig. 3b). In mouse neurons, we consistently observed a reduced TOM20 staining in areas having strong ps-αSyn staining (Fig. 3b, arrows), which was also observed in PFF-treated rat neurons (Additional file 1: Figure S5, arrows). We reasoned that the observation is likely due to the high aggregation propensity of mouse αSyn, which forms tightly packed aggregates and interferes with the detection of mitochondrial markers by immunofluorescence staining. To verify the co-localization of ps-αSyn and TOM20, we performed a proximity ligation assay (PLA) with antibodies against ps-αSyn and TOM20 in PFF-treated rat primary neurons. We found that positive signals were only detected in primary neurons treated with PFF (Fig. 3c), which supported our conclusion that the majority of PFF-induced ps-αSyn is associated with mitochondria.

### Aggregated αSyn, but not physiological αSyn, preferentially binds to mitochondria

**Fig. 4:**
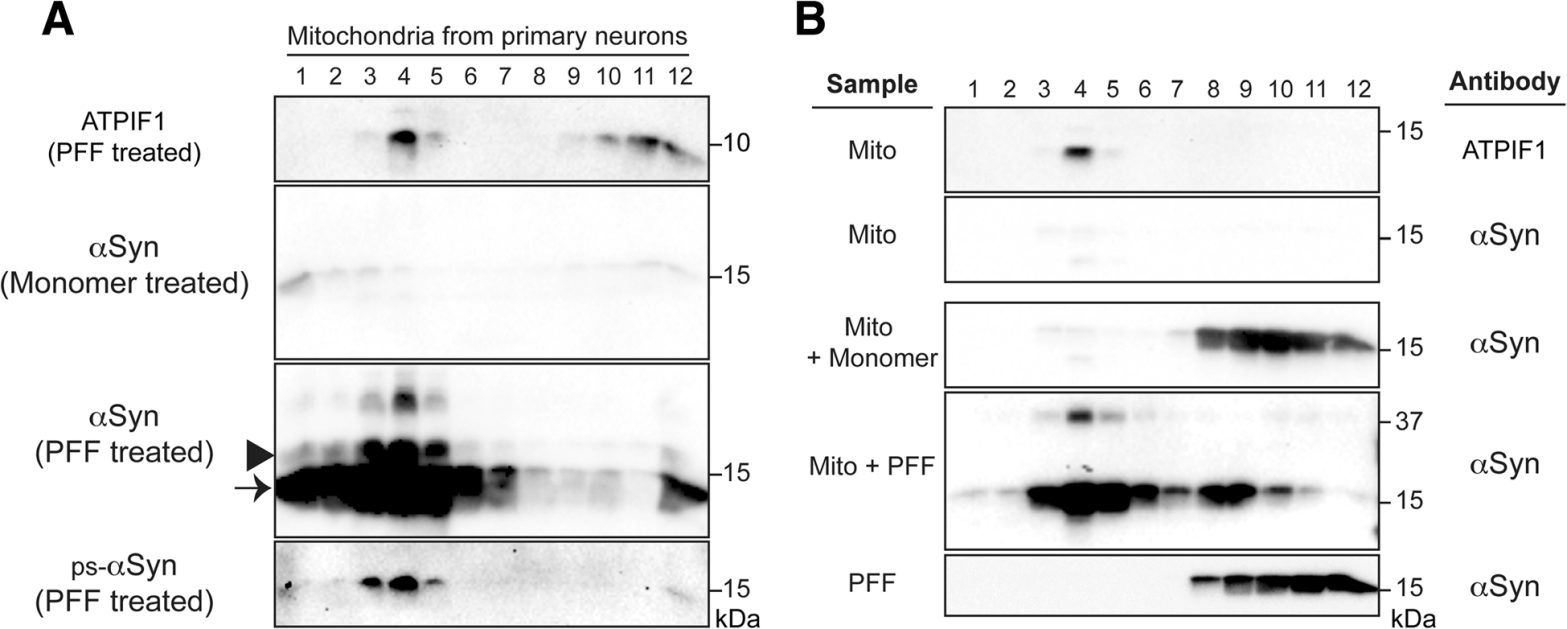
Aggregated αSyn preferentially binds to mitochondria. **a** Mitochondria isolated from αSyn monomer-treated or PFF-treated neurons were separated by iodixanol gradient, and the presence of mitochondrial protein ATPIF1, total αSyn, and ps-αSyn was visualized by immunoblot analyses as indicated. The arrowhead indicates the position of full-length αSyn; the arrow indicates the position of truncated αSyn. **b** Isolated mitochondria with or without in vitro incubation with αSyn monomer or PFF for 30 min at room temperature were subjected to iodixanol gradient separation. PFF alone was also subjected to gradient separation as a control. The presence of the mitochondrial ATPIF1 or total αSyn in each fraction was visualized by immunoblot analyses as indicated

To compare the amount of physiological αSyn monomer and pathogenic αSyn aggregates associated with mitochondria, we isolated mitochondria from neurons treated with αSyn monomer or PFF and then subjected them to a density gradient. As expected, mitochondria migrated to the upper fraction (Fig. 4a, top panel). Using mitochondria isolated from neurons treated with αSyn monomer, we found little αSyn associated with the mitochondria (Fig. 4a, second panel). However, in PFF-treated neurons, a significant amount of αSyn (both full-length and truncated) was in the mitochondrial fraction, which co-migrated with ps-αSyn (Fig. 4a, third and fourth panels). Some of the truncated αSyn might derive from exogenously added PFF because PFF was efficiently cleaved in these neurons (Fig. 1a). Nevertheless, this result suggested to us that both full-length and truncated αSyn aggregates can be associated with mitochondria and that ps-αSyn only reflects a portion of full-length αSyn.

The large amount of αSyn accumulated in the mitochondrial fraction of PFF-treated neurons suggested to us that aggregated αSyn may have a preference to bind to mitochondria. To test this possibility, we purified mitochondria from untreated primary neurons, incubated isolated mitochondria with αSyn monomer or PFF in vitro, and then subjected the mixture to the density-gradient analysis. The purified mitochondria contained very little endogenous αSyn (Fig. 4b, second panel), which is consistent with the idea that very little physiological αSyn binds to mitochondria under normal conditions. After incubation with αSyn monomer or PFF, the majority of αSyn monomer remained at the bottom, but the majority of αSyn PFF co-migrated with the mitochondrial protein ATPIF1 to the upper fractions (Fig. 4b, third and fourth panels). As a control, αSyn PFF alone (i.e. without incubation with mitochondria) was also subject to the gradient separation, and it remained at the bottom (Fig. 4b, bottom panel). Therefore, our results suggested that relative to αSyn monomer, aggregated αSyn preferentially binds to the mitochondria.

### Preferential binding of ps-αSyn to mitochondria in other neuronal and transgenic mouse models

**Fig. 5:**
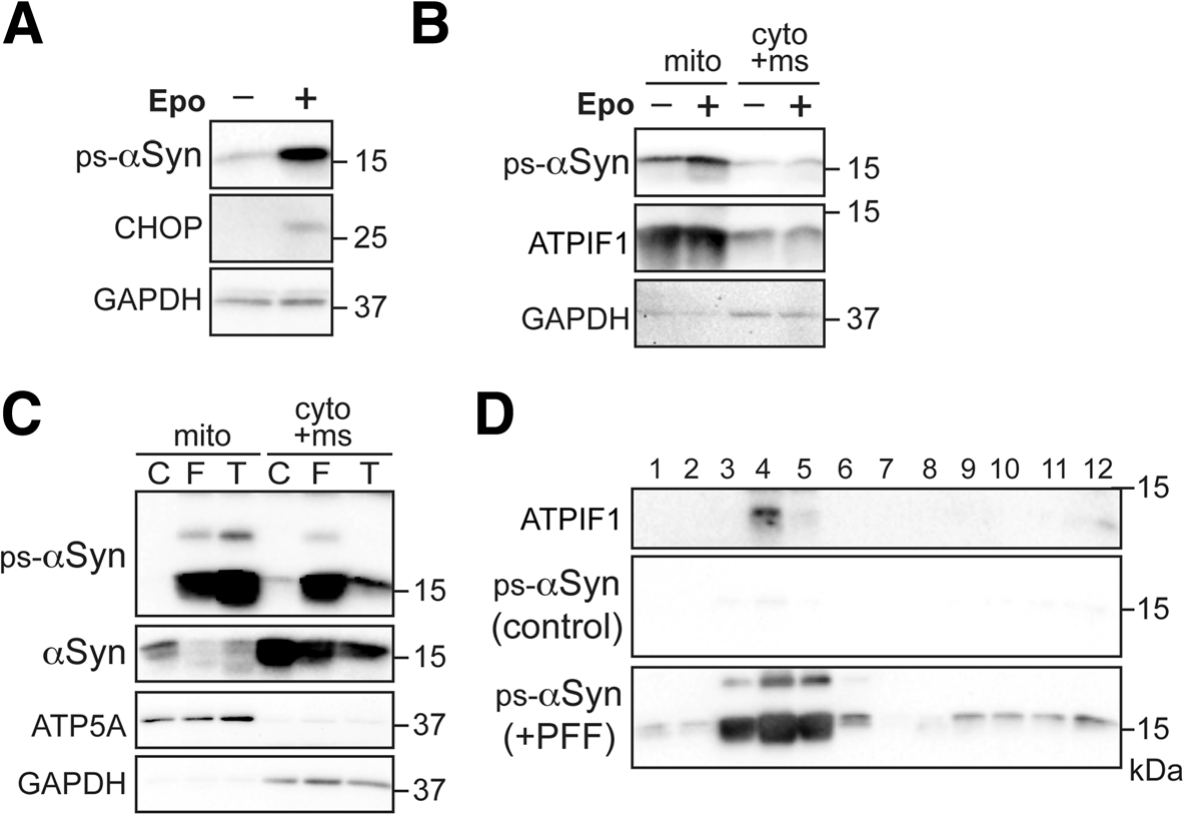
Association of ps-αSyn with mitochondria in primary neuron and transgenic mouse models. **a** ps-αSyn and CCAAT-enhancer-binding protein homologous protein (CHOP) accumulations in primary neurons treated with the proteasomal inhibitor epoxomicin (Epo) were determined by immunoblot analyses. **b** The homogenates prepared from Epo-treated (+) or untreated (−) neurons were separated into mitochondrial fraction (mito) and cytosolic/microsomal fraction (cyto+ms) by differential centrifugation. The mitochondrial enrichment of proteasomal-inhibition-induced ps-αSyn, mitochondrial ATPIF1, and the cytosolic marker GAPDH were detected by immunoblot analyses. **c** An A53T transgenic mouse was sacrificed at terminal stage of neurodegeneration (T, 305 d of age); Littermates of A53T transgenic mice intramuscularly injected with PBS (C) or αSyn PFF (F) were sacrificed when the PFF-injected mouse developed terminal neurodegeneration at 67 d after injection (135 d of age). Spinal cord homogenates prepared from these mice were separated into mitochondrial (mito) and cytosolic/microsomal (cyto+ms) fractions by differential centrifugation. ps-αSyn, total αSyn, mitochondrial ATP synthase (ATP5A), and cytosolic GAPDH were detected by immunoblot analyses. **d** Mitochondria isolated from the spinal cords of transgenic mice that received PBS (control) or αSyn PFF (+PFF) injections were subjected to iodixanol gradient separation, and the presence of ps-αSyn and ATPIF1 was detected by immunoblot analyses

The mitochondrial accumulation of ps-αSyn in PFF-treated neurons could be a result of the preferential mitochondrial binding by PFFs, which subsequently seed the conversion of endogenous αSyn on the surface of mitochondria. To rule out this possibility, we tested other neuronal and transgenic mouse models that do not require exogenously added PFF. First, we treated primary neurons with epoxomycin, a proteasome inhibitor, which is able to induce the accumulation of ps-αSyn (Fig. 5a) [40]. When the PNS prepared from epoxomycin-treated neurons were separated into mitochondrial and cytosolic/microsomal fractions, the ps-αSyn was enriched in the mitochondrial fraction (Fig. 5b). Second, we used a transgenic mouse model overexpressing human αSyn carrying the pathogenic A53T mutation [24]. This mouse model develops spontaneous neurodegeneration at old age with major pathological changes in the spinal cord and brain stem [30], and the disease can be significantly accelerated by intramuscular PFF inoculation [43]. Immunoblot analysis revealed a huge amount of ps-αSyn accumulated in the spinal cord of diseased mice, which was not present in young control mice that received PBS inoculation (Fig. 5c). Notably, regardless whether the disease occurred spontaneously or accelerated by PFF-inoculation, the ps-αSyn was mainly found in the mitochondrial fraction (Fig. 5c). In contrast, the majority of total αSyn, including the endogenous mouse αSyn and the αSyn from the human A53T αSyn transgene, was in the cytosolic/microsomal fraction (Fig. 5c). To rule out the possibility that the result was influenced by the aggregation status of ps-αSyn, we subjected the mitochondria fraction to the density-gradient analysis. The majority of ps-αSyn co-migrated with the mitochondrial marker ATPIF1 to upper fractions (Fig. 5d), confirming that ps-αSyn was indeed associated with mitochondria. Therefore, we concluded that the mitochondrial ps-αSyn accumulation is not due to exogenously added PFF, but is an inherent property of αSyn aggregates.

### The majority of ps-αSyn was associated with mitochondria in postmortem brain tissues from α-synucleinopathy patients

**Fig. 6:**
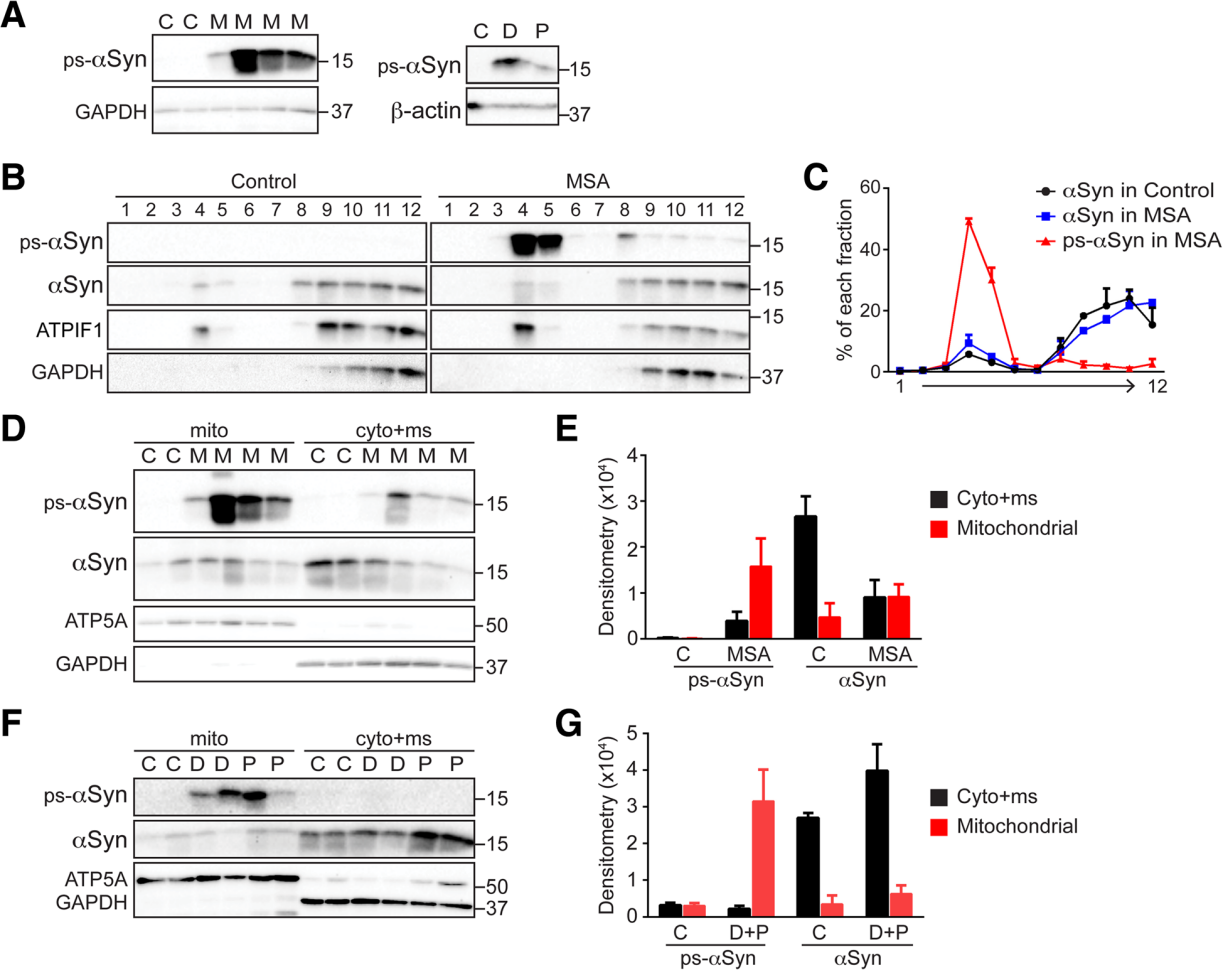
Association of ps-αSyn with mitochondria in α-synucleinopathy patients’ brain tissues. **a** ps-αSyn in brains of controls (C) and of MSA (M), DLB (D), and PD (P) patients was detected by immunoblot analysis; GAPDH or β-actin was used as a loading control. **b** PNS prepared from brains of controls or MSA patients were subjected to iodixanol gradient separation. The ps-αSyn, total αSyn, and ATPIF1 were detected by immunoblotting. **c** The curve reflects the percentage of ps-αSyn or αSyn in each fraction, which is the average ± standard error of two control samples and two MSA patient samples that were separated by the gradient individually. **d** PNS prepared from brains of controls (C) and MSA (M) patients were separated into mitochondrial (mito) and cytosolic/microsomal (cyto+ms) fractions, which were verified by immunoblot detection of mitochondrial ATP5A and cytosolic GAPDH. The ps-αSyn and total αSyn in each fraction were detected by immunoblot analysis. **f** The cingulate cortex of brains from controls (C), DLB patients (D), and PD (P) patients were subjected to the analyses described in panel **d**. Bar graphs in **e** and **g** reflect the quantification of data in panels **d** and **f**, respectively, which are average ± standard error of the amount of ps-αSyn and total αSyn in each fraction

To determine whether ps-αSyn is associated with mitochondria in patients’ brains, we tested postmortem patient brain tissues from various α-synucleinopathies (Additional file 1: Table S1). As expected, ps-αSyn was detected in the postmortem tissues from such patients, but not in tissue from controls (Fig. 6a). When PNS prepared from the striatum of MSA patient brains was subjected to the density-gradient separation, the majority of total αSyn remained at the bottom, similar to that of controls (Fig. 6b). The mitochondrial marker ATPIF1 migrated to the upper fractions, but a portion of it remained in the bottom, which could be due to the disintegration of some mitochondria in postmortem tissue. Remarkably, the majority of ps-αSyn migrated to upper fractions of the gradient and co-migrated with ATPIF1, which is completely different from total αSyn (Fig. 6b and c). When the homogenates from MSA patients were subjected to fractionation by differential centrifugation, the preferential enrichment of ps-αSyn in the mitochondrial fraction was obvious (Fig. 6d and e). Similar results were also observed with homogenates prepared from the cingulate cortex of PD and DLB patients (Fig. 6f and g). Together, these results suggest to us that in patients suffering from α-synucleinopathies, ps-αSyn was also preferentially associated with the mitochondria.

### Mitochondrial ps-αSyn accumulation is associated with defects in cellular respiration

**Fig. 7:**
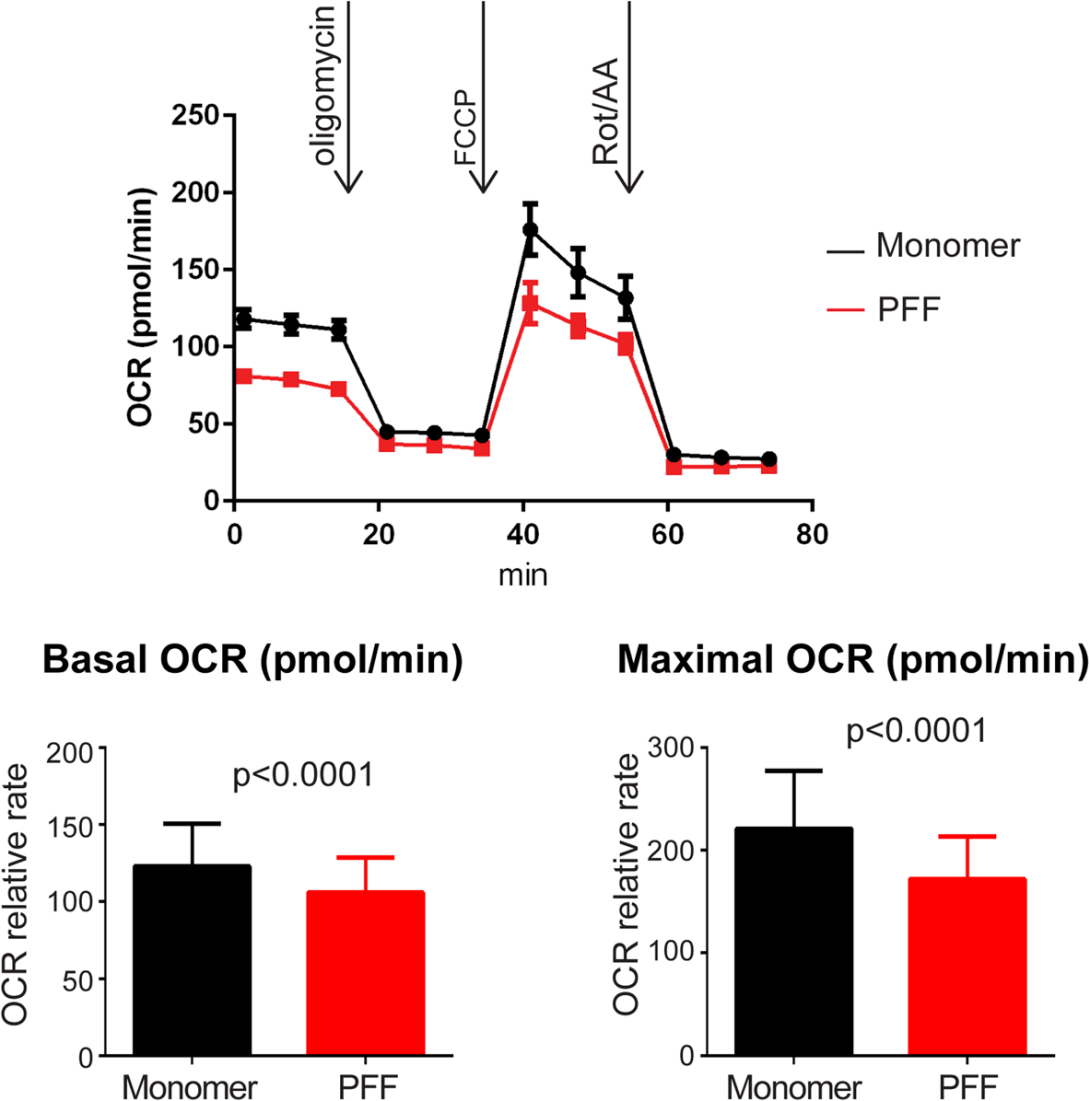
PFF treatment of primary neurons causes respiration defects. Mouse cortical neurons treated with αSyn monomer or PFF were subjected to the mitochondrial stress test measuring oxygen consumption rate. The differences in the basal and maximal respiration levels are shown in the bar graphs, which represent the average ± standard error of three independent experiments with 15–20 wells of cells per experiment. Statistical significance was determined by two-way ANOVA (for basal level, F = 33.66; p < 0.0001; for maximal level, F = 48.13; *P* < 0.0001)

To determine whether the ps-αSyn accumulation in the mitochondria affects mitochondrial function, we subjected the primary neurons treated with αSyn monomer or PFF to the mitochondrial stress test. The PFF-treated neurons consistently had a higher number of trifluoromethoxy carbonylcyanide phenylhydrazone (FCCP)-unresponsive wells (Additional file 1: Figure S6), indicating a weaker tolerance to FCCP treatment. More importantly, both the basal and maximum respiration capacities were significantly lower in PFF-treated neurons (Fig. 7), indicating that mitochondrial ps-αSyn accumulation does affect mitochondrial function.

## Discussion

Our results revealed a preferential accumulation of ps-αSyn in the mitochondria, connecting two major pathogenic events of α-synucleinopathies, i.e., αSyn misfolding and mitochondrial dysfunction. The fact that ps-αSyn from three different α-synucleinopathies was associated with mitochondria suggests to us that this is a general property of αSyn aggregates and is independent of the variable conformations of misfolded αSyn in different diseases [34]. Moreover, our results also showed that the majority of physiological αSyn is not associated with mitochondria, which is consistent with previous studies [5–7, 18, 19, 21] and suggests that the mitochondrial ps-αSyn accumulation is most likely not due to the phosphorylation of mitochondria-associated physiological αSyn. Instead, it is likely resulted from the inherent preference of pathogenic αSyn aggregates to bind to mitochondria, which is consistent with findings from our in vitro mitochondria binding study (Fig. 4b).

Our study also revealed that the mitochondrial ps-αSyn accumulation is accompanied by defects in mitochondrial activity. Together with the selective vulnerability of mitochondria in the energy-demanding nigral dopaminergic neurons [36, 48, 49], our findings may explain the fact that certain αSyn mutations or *SNCA* duplication cause disease similar to idiopathic PD [8] and that polymorphism of αSyn gene could be a risk factor for idiopathic PD [4]. Because the preferential binding by αSyn aggregates is not limited to mitochondria from nigral dopaminergic neurons, the ps-αSyn mitochondrial connection may also contribute to the degeneration of other neuronal types, which may lead to the cognitive and behavioral disturbances in DLB or PDD (Parkinson’s disease dementia) patients and in patients carrying *SNCA* triplication [32, 35, 45]. This conclusion is consistent with a recent report of fragmented mitochondria and clustering of αSyn aggregates in the mitochondria of neurons differentiated from human iPSCs carrying αSyn mutations [42] and the report of mitochondrial dysfunction in neurons differentiated from iPSCs derived from a patient carrying αSyn triplication [28].

Our findings also support the faithfulness of changes in PFF-induced models [29, 52] to the pathogenesis of α-synucleinopathies. The study of postmortem human tissues reflects the end stage of diseases, but the PFF-induced model allows us to study the dynamic cellular consequences of αSyn aggregation. Notably, some ps-αSyn did appear in the TBS-soluble fraction, and a large portion of it appeared in the Triton-soluble fraction (Fig. 1g), which presumably represents various states of polymerization, from a soluble oligomeric state to an amyloid fibril state. Because the majority of ps-αSyn co-migrated with mitochondria (Fig. 2b and c), it is reasonable to predict that not only αSyn aggregates in the fibril state, but also some of the αSyn oligomers, bind to mitochondria as well.

**Fig. 8:**
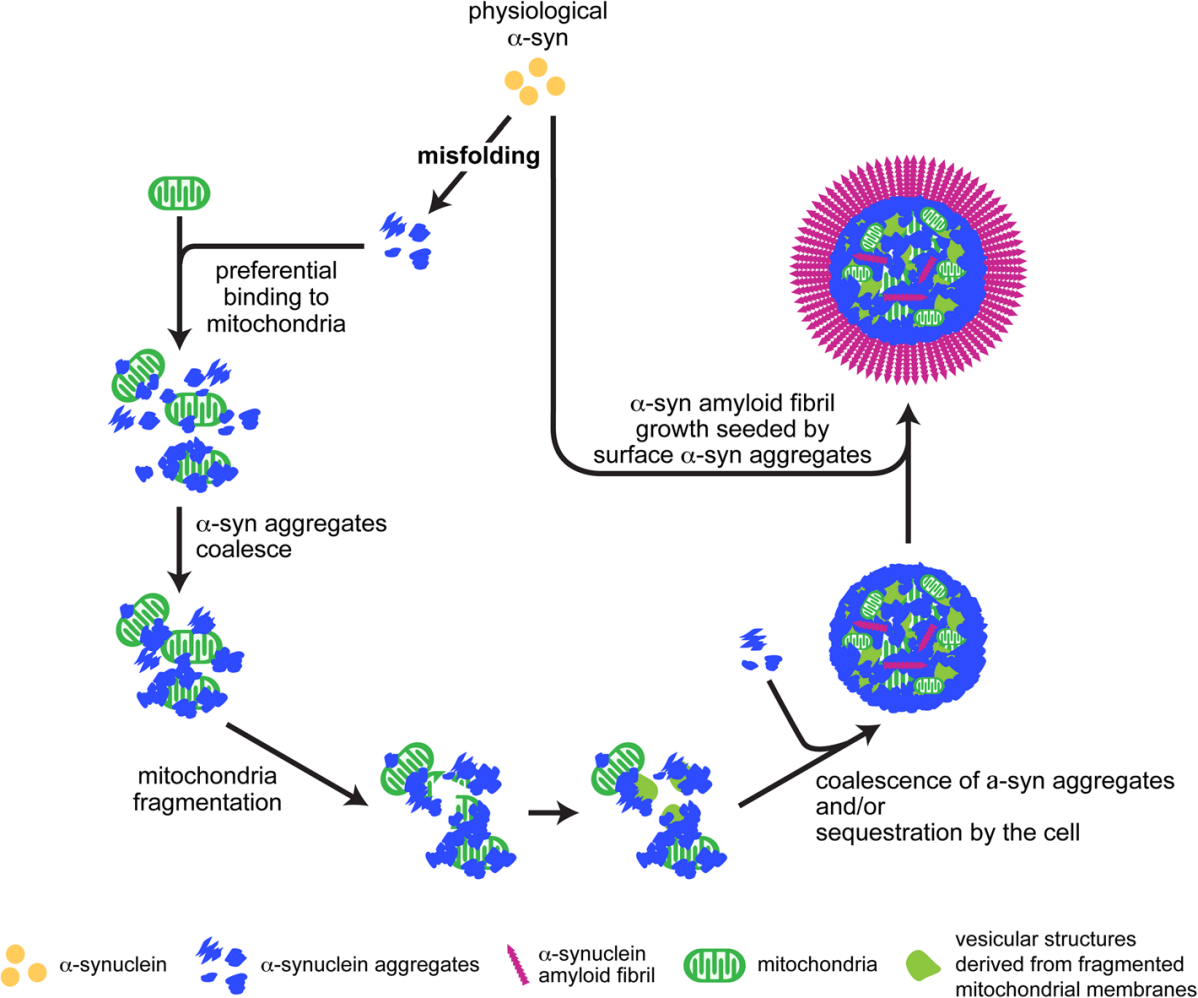
A schematic model of Lewy body formation

Because of the intrinsic tendency of αSyn aggregates to coalesce [23], binding of αSyn aggregates to mitochondria is not the end, but rather the middle, of a dynamic process (Fig. 8). The mitochondria-bound αSyn aggregates may further coalesce and/or the protein aggregate-bound mitochondria may be subjected to cellular clearance system. Thus, it is plausible to postulate that mitochondrial dysfunction could result from the accumulation of αSyn aggregates on or in mitochondria, which may interact with mitochondrial proteins and interfere with their normal functions. Alternatively, mitochondrial dysfunction may result from the tendency to coalesce by mitochondria-bound αSyn aggregates, which will disturb the normal mitochondria movement, dynamics, and morphology. When αSyn aggregation becomes overwhelming, it could also disrupt mitochondria, resulting in fragments of mitochondrial membranes (Fig. 8). This process, together with cell’s ability to concentrate misfolded proteins [22], may ultimately lead to the formation of Lewy Bodies.

It is important to note that the mitochondria-associated αSyn aggregates remain competent to seed αSyn monomer to form ordered aggregates. While αSyn aggregates in the middle of Lewy bodies are either already coalesced without reactive surface or crowded by membranous structures without the access to αSyn monomer, the αSyn aggregates on the surface remain competent to seed and have the accessibility to αSyn monomer (Fig. 8). Thus, in neurons where αSyn monomer is available, αSyn amyloid fibrils can be grown on the surface of Lewy bodies, resulting in a ring of densely packed αSyn fibrils that reacts strongly with antibody during immunohistochemical staining [31]. Therefore, our model predicts that at the end stage, Lewy bodies or Lewy neurites may contain membranous structures, mitochondria, αSyn aggregates in complex with mitochondria and vesicles, and αSyn fibrils [31, 39, 46].

## Conclusion

Our study provides solid evidence supporting the preferential binding of pathogenic αSyn species to mitochondria; suggests mitochondrial dysfunction as the common downstream pathogenic mechanism for αSyn aggregation; and points out that disrupting the binding of pathogenic αSyn species to mitochondria could be beneficial to patients suffering from α-synucleinopathies.

## Additional file

Additional file 1: **Table S1.** Postmortem tissues**. Figure S1.** Electron microscopic images of αSyn PFF before and after sonication. **Figure S2.** PFF-induced ps-αSyn in rat neurons. Primary cortical neurons were untreated (−) or treated with αSyn monomer (M) or PFF (F) as indicated. The presence of ps-αSyn was detected by immunofluorescence staining (red), and MAP2 stain (green) was used as a neuronal marker. Nuclei were stained with DAPI. The lower left panel shows a representative immunoblot of ps-αSyn and total αSyn; immunoblot analysis of GAPDH was performed to verify equal loading. The bar graph represents the average ± standard error of five independent experiments. Statistical significance was determined by one-way ANOVA followed by a Dunnett’s multiple comparison test (F = 51.21, *n* = 5, *p* < 0.0001). **Figure S3.** Postnuclear supernatant (PNS) prepared from PFF-treated primary neurons was separated using a discontinuous sucrose gradient, and fractions were collected from the top to the bottom. The presence of ps-αSyn, mitochondrial ATPIF1, calnexin (endoplasmic reticulum), and syntaxin 6 (Golgi) in each fraction were detected by immunoblot analysis. **Figure S4.** PFF-induced ps-αSyn accumulated in the mitochondria of mouse primary neurons. PNS of mouse primary neurons treated with either αSyn monomer (M) or PFF (F) were separated into mitochondrial (mito) and cytosolic/microsomal (cyto+ms) fractions. The presence of ps-αSyn, mitochondrial ATP synthase (ATP5A), and cytosolic GAPDH was detected by immunoblot analysis. **Figure S5.** Immunofluorescence staining was performed on PFF-treated rat primary neurons with antibodies against ps-αSyn and TOM20 as indicated. Arrows indicate cellular areas with strong ps-αSyn stain, but weak TOM20 stain. The top and bottom panels are two separated images. **Figure S6.** PFF-treated neurons were less responsive to FCCP treatment. The graph represents the average ± standard error of four independent experiments. The statistic difference was determined by paired *t*-test (*p* = 0.0029; *n* = 4). (DOCX 1025 kb)
