# Supplementary material for: Pathogenic alpha-synuclein aggregates preferentially bind to mitochondria and affect cellular respiration
Source: Acta Neuropathol Commun. 2019 Mar 14;7:41. doi: 10.1186/s40478-019-0696-4 (PMC6419482; doi:10.1186/s40478-019-0696-4)
Supplement: Supplementary file 1 — Table S1. Postmortem tissues. Figure S1. Electron microscopic images of αSyn PFF before and after sonication. Figure S2. PFF-induced ps-αSyn in rat neurons. Primary cortical neurons were untreated (−) or treated with αSyn monomer (M) or PFF (F) as indicated. The presence of ps-αSyn was detected by immunofluorescence staining (red), and MAP2 stain (green) was used as a neuronal marker. Nuclei were stained with DAPI. The lower left panel shows a representative immunoblot of ps-αSyn and total αSyn; immunoblot analysis of GAPDH was performed to verify equal loading. The bar graph represents the average ± standard error of five independent experiments. Statistical significance was determined by one-way ANOVA followed by a Dunnett’s multiple comparison test (F = 51.21, n = 5, p < 0.0001). Figure S3. Postnuclear supernatant (PNS) prepared from PFF-treated primary neurons was separated using a discontinuous sucrose gradient, and fractions were collected from the top to the bottom. The presence of ps-αSyn, mitochondrial ATPIF1, calnexin (endoplasmic reticulum), and syntaxin 6 (Golgi) in each fraction were detected by immunoblot analysis. Figure S4. PFF-induced ps-αSyn accumulated in the mitochondria of mouse primary neurons. PNS of mouse primary neurons treated with either αSyn monomer (M) or PFF (F) were separated into mitochondrial (mito) and cytosolic/microsomal (cyto+ms) fractions. The presence of ps-αSyn, mitochondrial ATP synthase (ATP5A), and cytosolic GAPDH was detected by immunoblot analysis. Figure S5. Immunofluorescence staining was performed on PFF-treated rat primary neurons with antibodies against ps-αSyn and TOM20 as indicated. Arrows indicate cellular areas with strong ps-αSyn stain, but weak TOM20 stain. The top and bottom panels are two separated images. Figure S6. PFF-treated neurons were less responsive to FCCP treatment. The graph represents the average ± standard error of four independent experiments. The statistic difference was determined by paired t- [file 40478_2019_696_MOESM1_ESM.docx]

**Electronic supplemental materials**

**Supplemental table.** Postmortem tissues

| **Brain Bank**  **ID** | **Age at death**  **(years)** | **Sex** | **Postmortem delay (h)** | **Neuropath. diagnosis** | **Sample area** |
| --- | --- | --- | --- | --- | --- |
| 1432 | 83 | Female | 21 | Control | Striatum |
| 14 | 65 | Male | 24 | Control | Cingulate cortex |
| 172 | 70 | Male | 15 | Control | Striatum & cingulate cortex |
| 1903 | 64 | Male | 68 | MSA | Striatum |
| 1860 | 63 | Female | 13 | MSA | Striatum |
| 1841 | 57 | Male | 21 | MSA | Striatum |
| 1836 | 61 | Male | 5 | MSA | Striatum |
| 1665 | 66 | Male | 15 | DLB without AD | Cingulate cortex |
| 1076 | 82 | Female | 10 | DLB without AD | Cingulate cortex |
| 824 | 86 | Male | 10 | PD | Cingulate cortex |
| 125 | 81 | Male | 16 | PD | Cingulate cortex |

**Supplemental figures**


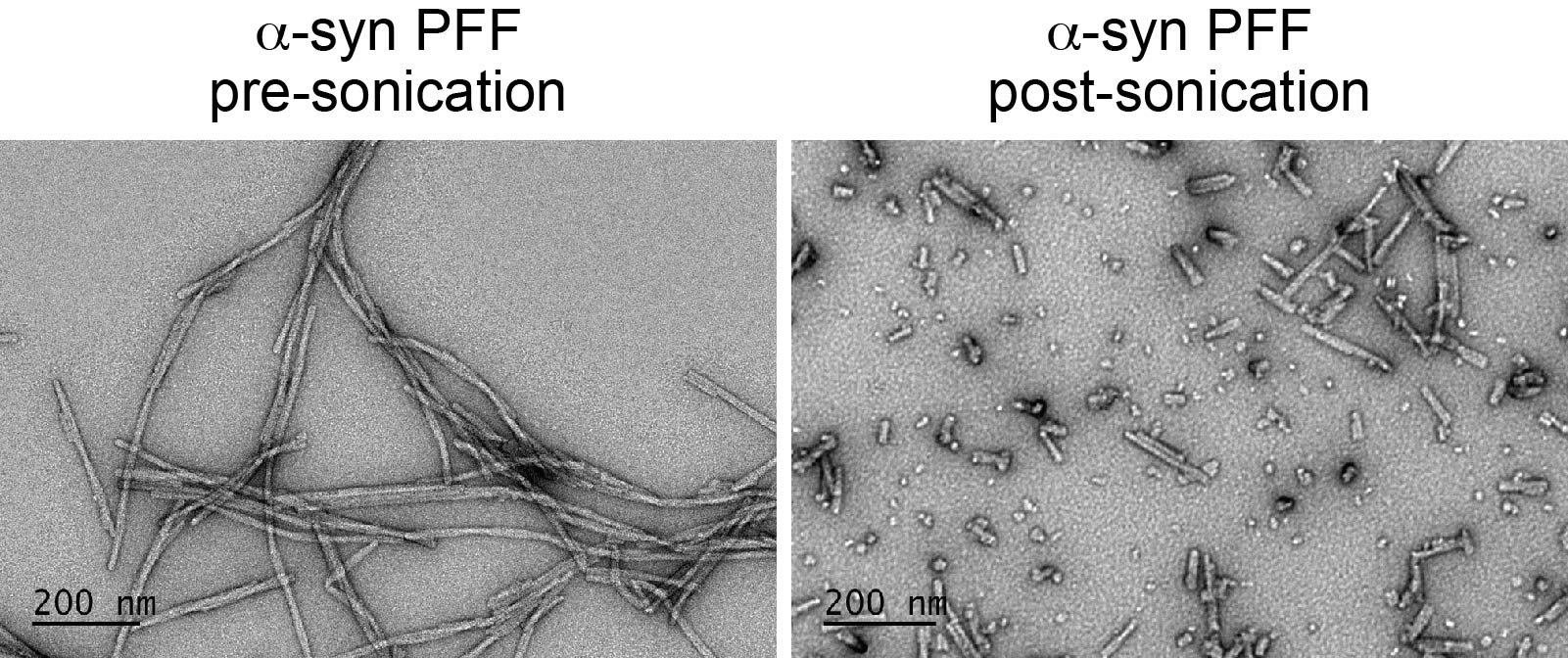


**Figure S1.** Electron microscopic images of αSyn PFF before and after sonication.


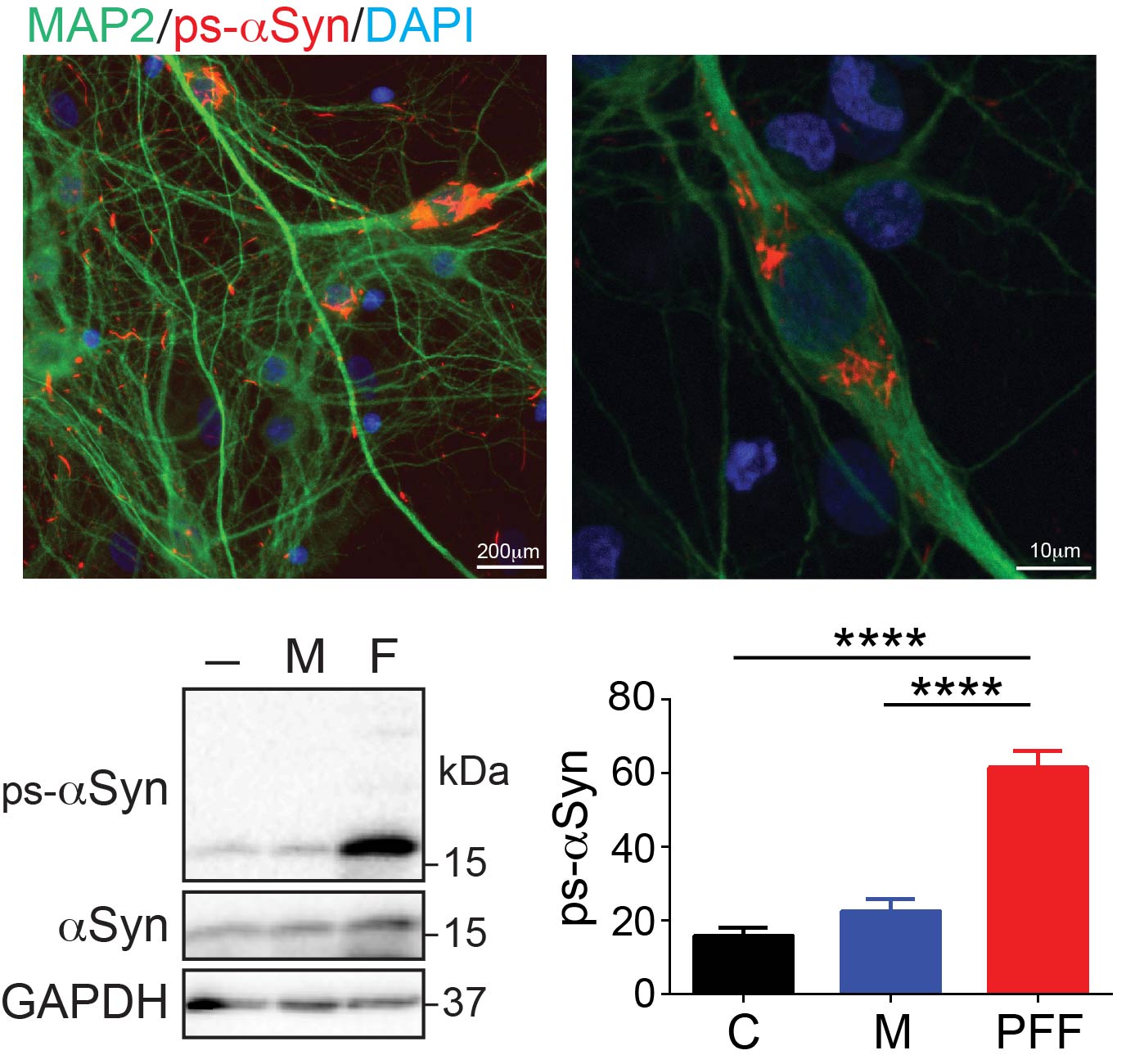


**Figure S2.** PFF-induced ps-αSyn in rat neurons. Primary cortical neurons were untreated (–) or treated with αSyn monomer (M) or PFF (F) as indicated. The presence of ps-αSyn was detected by immunofluorescence staining (red), and MAP2 stain (green) was used as a neuronal marker. Nuclei were stained with DAPI. The lower left panel shows a representative immunoblot of ps-αSyn and total αSyn; immunoblot analysis of GAPDH was performed to verify equal loading. The bar graph represents the average ± standard error of five independent experiments. Statistical significance was determined by one-way ANOVA followed by a Dunnett’s multiple comparison test (F = 51.21, n =5, p < 0.0001).

**
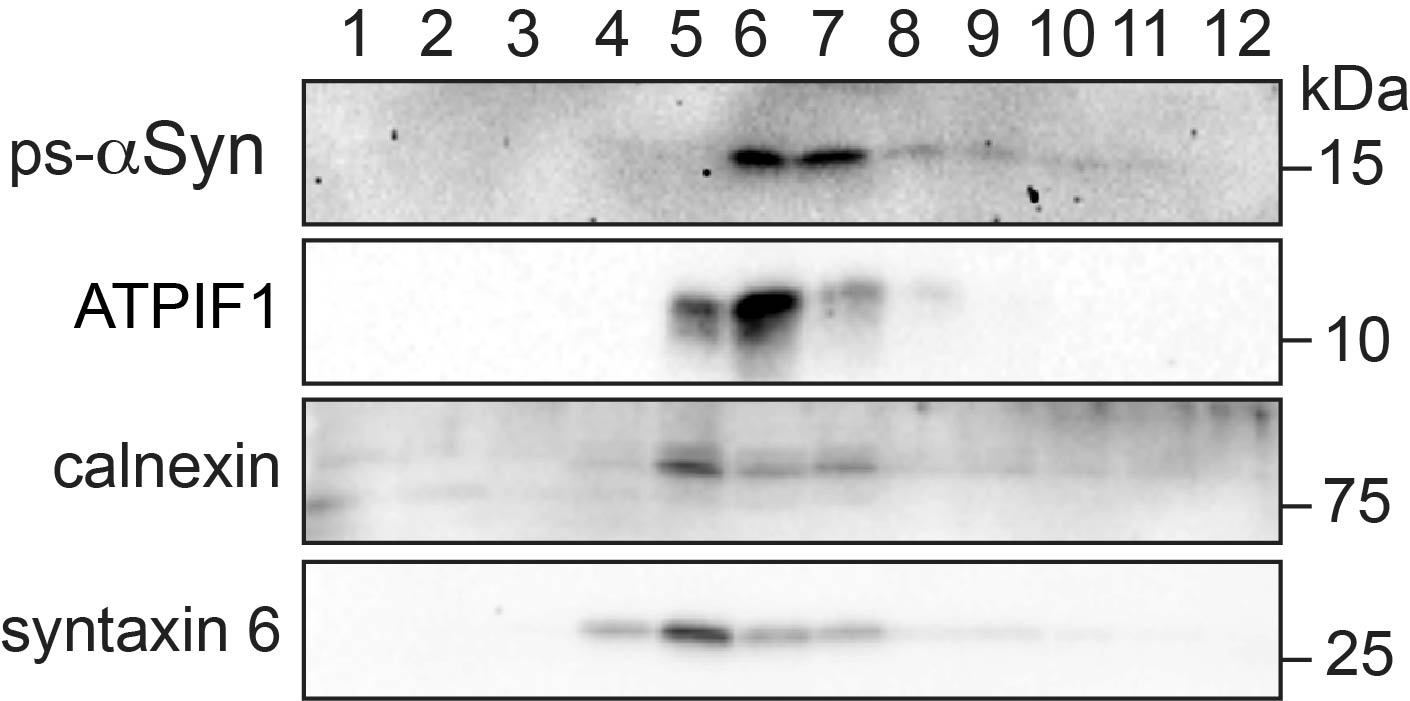
**

**Figure S3.** Postnuclear supernatant (PNS) prepared from PFF-treated primary neurons was separated using a discontinuous sucrose gradient, and fractions were collected from the top to the bottom. The presence of ps-αSyn, mitochondrial ATPIF1, calnexin (endoplasmic reticulum), and syntaxin 6 (Golgi) in each fraction were detected by immunoblot analysis.


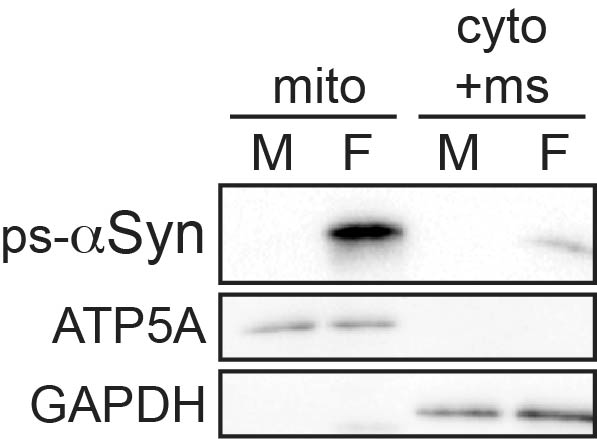


**Figure S4.** PFF-induced ps-αSyn accumulated in the mitochondria of mouse primary neurons. PNS of mouse primary neurons treated with either αSyn monomer (M) or PFF (F) were separated into mitochondrial (mito) and cytosolic/microsomal (cyto+ms) fractions. The presence of ps-αSyn, mitochondrial ATP synthase (ATP5A), and cytosolic GAPDH was detected by immunoblot analysis.


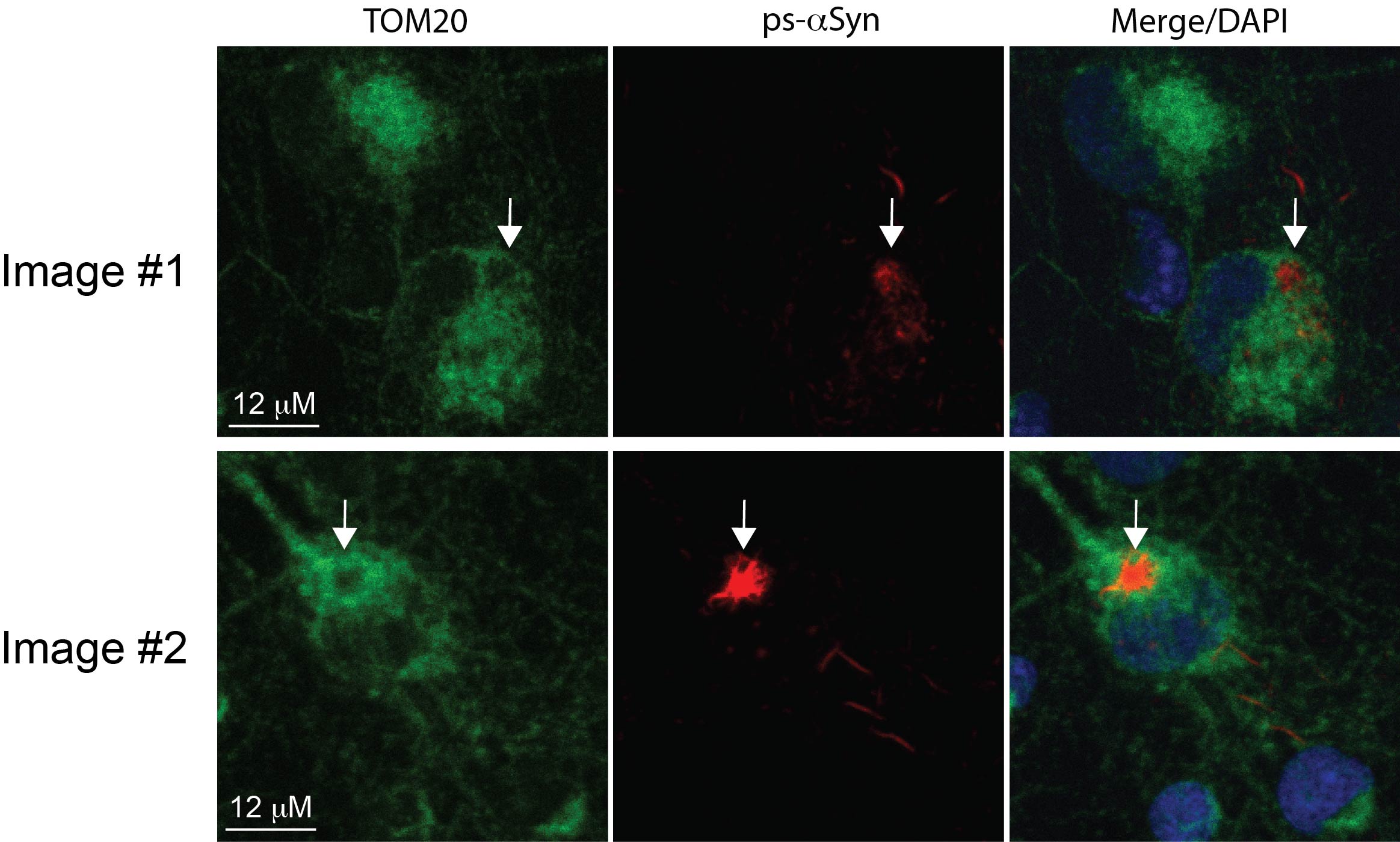


**Figure S5.** Immunofluorescence staining was performed on PFF-treated rat primary neurons with antibodies against ps-αSyn and TOM20 as indicated. Arrows indicate cellular areas with strong ps-αSyn stain, but weak TOM20 stain. The top and bottom panels are two separated images.

**
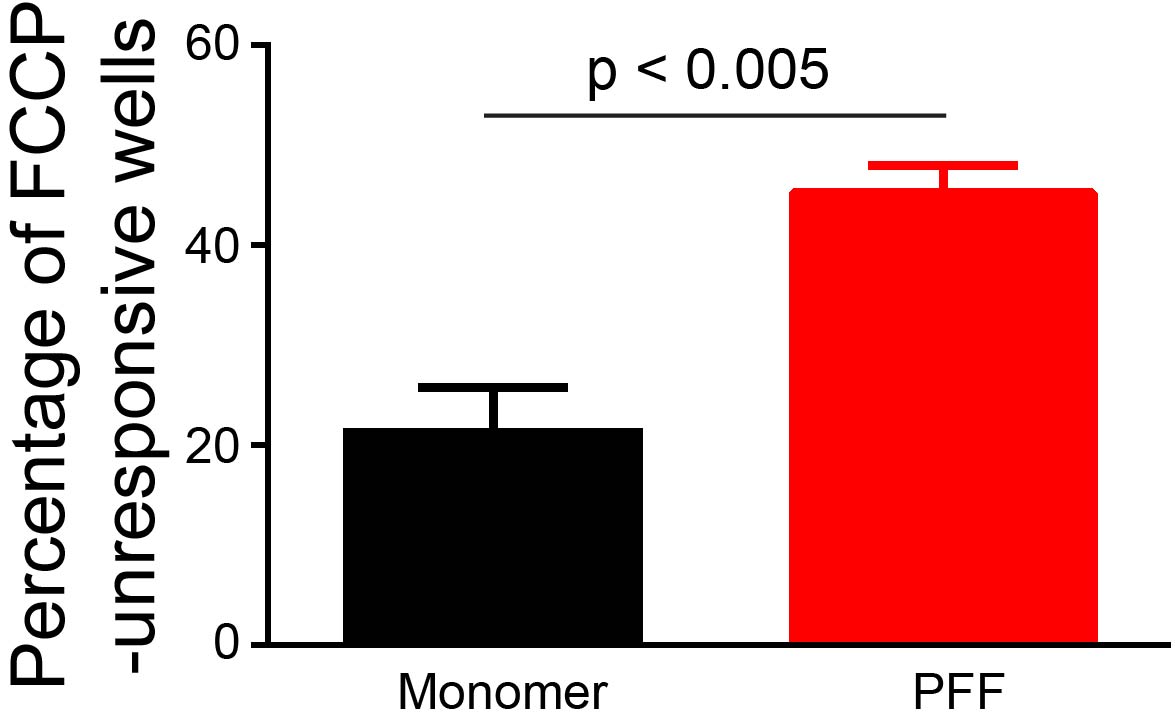
**

**Figure S6.** PFF-treated neurons were less responsive to FCCP treatment. The graph represents the average ± standard error of four independent experiments. The statistic difference was determined by paired *t*-test (p = 0.0029; n = 4).
